# Partially divided caging reduces overall aggression and anxiety which may indicate improved welfare in group housed male C57BL/6J mice

**DOI:** 10.1186/s12917-024-03918-y

**Published:** 2024-02-24

**Authors:** Bret R. Tallent, L. Matthew Law, Jonathan Lifshitz

**Affiliations:** 1 Neurotrauma and Social Impact research team, Department of Psychiatry, University of Arizona College of Medicine – Phoenix, BSPB Building, 475 N. 5th Street, Phoenix, AZ 85004 USA; 2 Phoenix Veterans Affairs Health Care System, Phoenix, AZ USA

**Keywords:** Welfare, Mouse, Aggression, Fighting, Anxiety

## Abstract

Deciding which environmental enrichment is used in mouse caging is often subjective, with cost frequently prevailing over welfare benefits, including aggression and anxiety. While many devices introduced to encourage natural behaviors and reduce aggression show mixed results, we have previously demonstrated significant reductions in aggressive behavior between group-housed male mice housed in partially divided caging. To further assess behavior, we have raised male C57BL/6J mice in either partially divided caging or in standard caging with no divider. Animal behavior was tested on rotarod, open field, novel object recognition, elevated plus maze, and Y maze. Body weights were taken weekly beginning at weaning and bite wounds were counted weekly beginning at 133 days old. Aggressive behavior was recorded weekly beginning at 133 days old. Results indicated significantly less anxiety in the elevated-plus maze, statistically fewer bite wounds, and a statistically significant decrease in aggressive behaviors of mice in partially divided caging compared to mice in standard cages. We conclude that reductions in anxiety, aggressive behavior, and bite wounds may indicate improved overall welfare for non-sibling, group housed male mice.

## Introduction

In nature, mice typically live burrowed in demes [1, 2]. Deme size is dependent upon available resources in the territory, with larger demes formed in areas with abundant resources [3–7]. A dominant male incorporates several females and subordinate males, while protecting against unfamiliar males. Aggressive interactions with unfamiliar males escalate until proper social cues are given (e.g. avoiding line of sight or leaving) [8–10]. Larger demes lead to more aggressive events, and safety is often sought in the burrows. In the laboratory, wherein cage size and density are regulated [11], the ‘territory’ is maximized for animal housing. While resources are adequate, retreating to safety and social behavioral responses are reduced. In a laboratory cage, opportunities to express innate behavior to social cues, such as breaking line of sight, are extremely limited, which develops a dysfunctional dominance hierarchy [4, 5, 12–22] and compromises deme welfare [18, 23]. Therefore, enrichment objects are necessary to establish a more natural environment and promote natural dominance-subordinate behaviors.

Enrichment objects increase cage complexity and encourage natural expression of behavior [24–27], however considerable debate continues regarding the effectiveness on mouse physiology and behavior [9, 12, 16, 18, 22, 25, 28]. Individual items added to a cage are a limited resource that can evoke territorialism and aggression, or allow for ambush behaviors (e.g., huts, tubes, lofts, structures) [1, 21, 24, 26, 29–34]. Too many objects obscure observation of the animals, requiring personnel to open cages for health and wellness checks. Alternatives to enrichment objects include complex caging and dividers for mice [7, 12, 25, 31, 35].

In nature, mice burrow to reduce exposure to the elements, evade environmental dangers, and escape from threats [25]. Chamove et al. (1989) reports that “mice reared in a complex cage system that emulates a burrow-like environment are healthier and less reactive compared to those in standard housing.” More recently, Cait et al. (2022) reported results of a meta-analysis confirming that standard caging has adverse effects on animal welfare [36]. Physiological and behavioral benefits included lower adrenal weight, increased body weight, and increased activity compared to mice in a standard cage [25]. Based on our pilot study [35], dividers effectively increase cage complexity by dividing half of the home cage into thirds. Increased wall space facilitated thigmotactic preferences of rodents without compromising floor space [37], or obscuring visualization of animals. While divided caging has shown statistically significant decreases in aggressive behavior [35] and improvements in overall health [25], the chronic impact of divided caging on overall aggression, welfare benefits, and possible behavioral changes has not been explored.

The study objectives were to validate and expand upon previous results to include neurological behavioral testing, body weights, and bite wounds in the C57BL/6J strain. We hypothesized that partial cage dividers would decrease overall aggression and reduce anxiety, indicating improved welfare of group-housed mice. We first performed standard neurological and anxiety tests to determine overt changes in behavior that might affect scientific rigor or health and welfare as mice aged through 90 days old. To maximize the utility of these mice, a second study was started at 133 days old to quantify aggression between mice as contact encounters and bite wound counts. As a cross-over study design, the introduction or removal of the cage-divider would demonstrate an enduring hierarchy and potential for late intervention to quell aggression.

## Materials and methods

### Study design

**Fig. 1:**
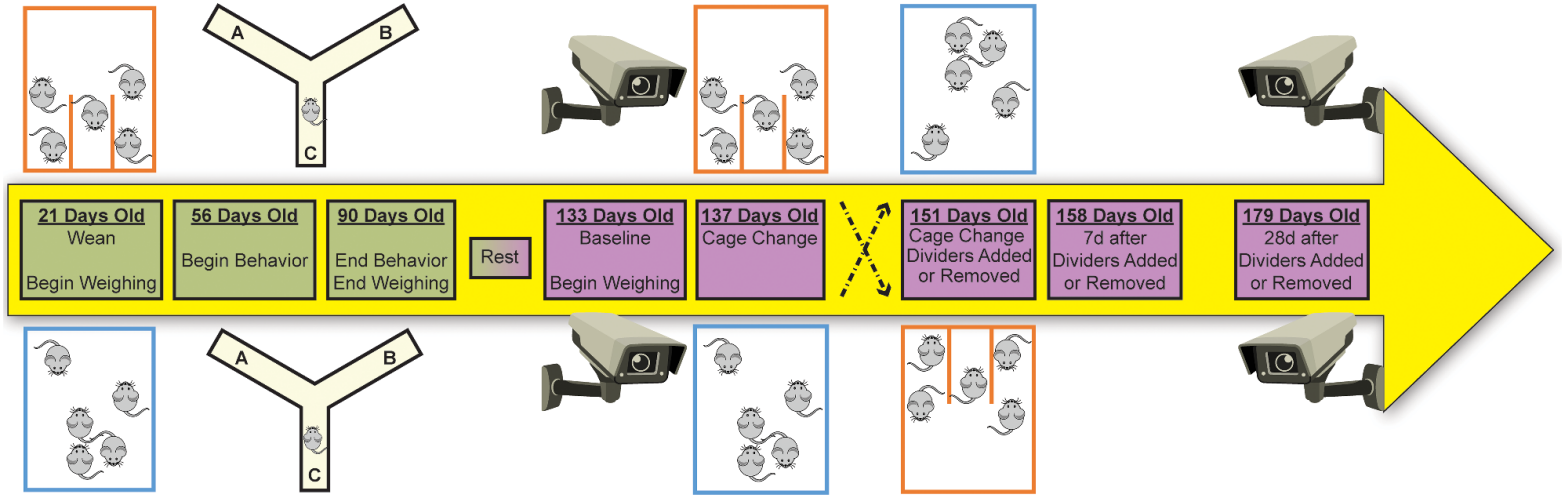
Timeline beginning at 21 days old. Weekly weights collected at 28–90 days old and 133–180 days old. Behavioral assessments began at 56 days old and continued through 90 days old. Following 42 days of “rest” to age the animals, home cage behavior was recorded and bite wounds counted. At 133 days old, aggression observations served as baseline for the remainder of the study. In a cross-over study design, the cage divider was removed from divided cages or a new divider was introduced to standard cages at 151 days old during a cage change. Both 7 day and 28 day after the removal or introduction of a cage divider were recorded between cage changes

The two objectives were accomplished using the same set of mice to adhere to the reduction principle of in vivo experimentation (Fig. 1). Mice housed in standard or partially-divided cages were evaluated for body weight growth and neurobehavioral performance through 90 days old. In a cross-over study design, after 43 days undisturbed, the level of aggression was recorded as physical encounters and bite wounds before and after a cage-divider was either introduced or removed from specific cages. Mice were used to teach animal handling for the remainder of their lives following the conclusion of this study.

### Animals

All animal studies were conducted in accordance with guidelines established by the internal IACUC (Institutional Animal Care and Use Committee) at the University of Arizona and NIH guidelines for the care and use of laboratory animals. Studies are reported following ARRIVE (Animal Research: Reporting In Vivo Experiments) guidelines [38]. Male mice were studied due to standard procedures of same sex housing and higher male mouse aggression toward conspecifics than female mice. Pre-determined exclusion and humane endpoint criteria included removing any animal from study that had visible wounds requiring veterinarian intervention (skin lesion ≥ 5 mm diameter); no animals were excluded from study. Five C57BL/6J dams with 10 male pups each (*n* = 50 mice) were shipped from a commercial vendor (The Jackson Laboratory, Bar Harbor, Maine) and acclimated in standard individual ventilated caging before weaning at 21 days old. The 50 pups would become the study animals upon weaning. Prior to shipment, the vendor fostered pups from various dams to provide 1 dam with 10 male pups at 14 days old. Dams with pups remained in the vivarium for 7 days and acclimated in standard IVC caging (Innocage^®^ Mouse Cage, Innovive, San Diego, CA) before weaning. Randomization of animals was achieved by weaning a single animal from each dam into one of 10 cages to ensure equal distribution across two caging conditions. Mice were weaned into groups of five and housed in either standard or divided caging (*n* = 5 cages per caging condition). Two phases to this report (behavior and body weights, then aggression) took advantage of reduction principles of in vivo animal use and required two different outcome measures and a gap between measurements. In phase one, body weight and behavior was assessed at the level of single animals (*n* = 50). In phase two, aggression was measured at the level of cages (*n* = 10; 5 divided, 5 non-divided). All mice were handled in a similar way to avoid any between group confounding effects. Animals were handled by the tail and by the same technician each time. Nesting material was transferred into clean cage at time of cage changes to sustain welfare between animals [39].

### Housing

Mice were housed in a 14 h light/10 h red light (Light Gard Light Tubes, Solar Graphics, Clearwater, FL) cycle at a constant temperature (23 ± 2°C) and humidity (50% ±10%). White light intensity at cage level was 48 lumens, and red light intensity was 4 lumens. Food (Teklad 2919 irradiated, Envigo, Placentia, CA) and water (Innovive pre-filled acidified water) were available *ad libitum*. Cages were sterile and came pre-bedded with 1/8” corncob bedding. All cages contained a single water bottle, feed hopper, corncob bedding, and a nesting square (Ancare Nestlets, Belmore, NY) [28]. One nesting square can reduce thermal distress [40] without obscuring the view to assess aggressive behavior videos. Dividers were added to standard cages with no other modifications in housing conditions. Routine husbandry included daily evaluation and documentation of each animal’s condition by hand, by the same animal caretaker throughout the duration of the study. Treatment of wounds was not required. Cage changes occurred every other week, however cage dividers were not replaced and remained with the animals throughout study as detailed in the timeline (Fig. 1). The cross-over study design was implemented over the final 34 days of the study, where cage dividers were introduced to or removed from the cages.

### Cage dividers

**Fig. 2:**
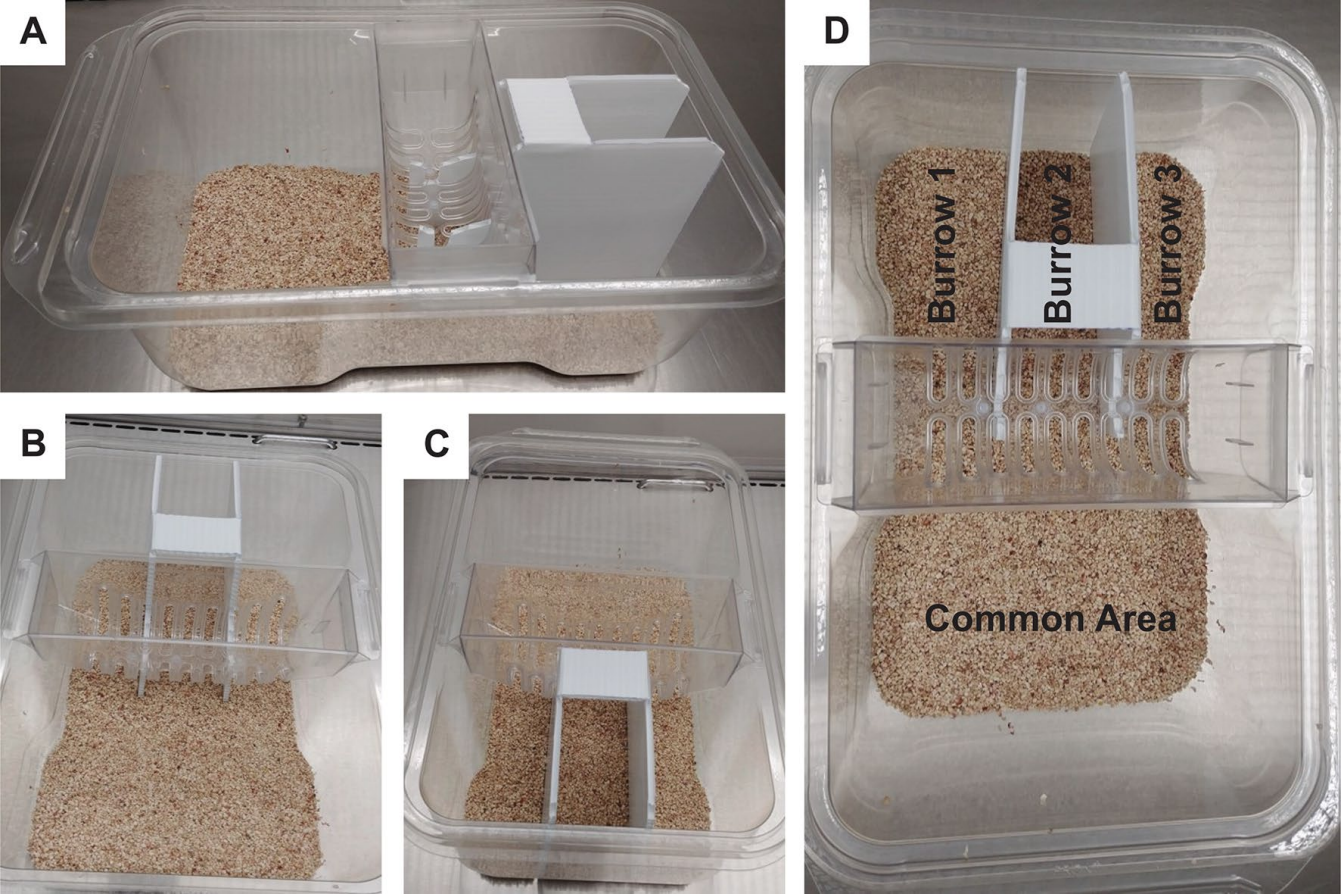
Standard Innovive cage with a partial cage divider, as seen from side (**A**), front (**B**), rear (**C**), and top (**D**). Half the cage is divided into thirds allowing full access to all areas by all animals. Multiple walls appeal to thigmotactic nature of mice and provide avenues for subordinates to break line of sight of dominant animals

Cage dividers were hand-fabricated from opaque, white, corrugated plastic (1.6 mm thick B flute from sheets that divided the levels of water bottles on the shipping pallet from Innovive) and sterilized with vaporized hydrogen peroxide prior to introduction into cages (37.3 × 23.4 × 14.0 cm). Designs allowed dividers to fit inside existing cages without modification to enclosures or obstructing ventilation (Fig. 2A). Dividers were held in place by the feed hopper and cage lid (Fig. 2B and C), effectively dividing half of the cage into thirds (Fig. 2D), from cage floor to cage lid. Approximately 3 mm of cage width was reduced by the cage dividers and added approximately 74.6 cm of wall length. All four compartments (common area and three burrows) were open to one another and accessible. Total floor space (522.58 sq cm^2^) and head room was unaffected by the addition of the divider. The walls of the divider ran parallel to the long axis of the cage, maintaining line-of-sight for daily observation and welfare checks by animal care personnel.

### Behavioral tests

Standard neurological and anxiety tests were used to assess overt changes in behavior that might affect scientific rigor or health and welfare of the animals. These tasks included: rotarod to test motor function and fine balance, open field and elevated plus maze tests for anxiety and exploratory behavior, novel object recognition (NOR) to assess recognition memory, and the Y-maze to assess spatial navigation strategies. To adjust for differences in behavioral profiles, two assays were used for each general behavior of interest. For motor, rotarod and open field was used. For cognition, novel object and y-maze was used. For anxiety, elevated plus and open field was used. White light intensity at test level for each test was between 50 and 56 lumens.

Behaviors were assessed over 34 days beginning at 56 days old. Though mice are considered sexually mature at 35 days old, they are not considered full adults until 90 days old. We chose 56 days old to encapsulate young adult to adult phases of behavior and avoid juvenile behavioral changes. This timeline is based upon The Jackson Laboratory website (https://www.jax.org/research-and-faculty/research-labs/the-harrison-lab/gerontology/life-span-as-a-biomarker). As the cage divider is a physical object, it was impossible to blind the handler to the group conditions as the mice were taken from cages with either the divider present or not.

Cages of animals were alternated during testing to avoid order effect. All behavioral equipment was cleaned with quaternary ammonia followed by 7% hydrogen peroxide between trials, and air-dried for five minutes. Only one behavioral test was performed on a given day. For all behavioral tests, except rotarod (computer timed), Ethovision software (Noldus, Leesburg, VA) was used to record and assess behavioral measures for each task and remove bias from the assessment. There was no live scoring of behavioral tests. One male and one female ran the behavior tests with the female experimenter handling all animals, but the male was in the room to assist with set up and clean up.

### Rotarod (Rotamex, Columbus instruments)

Assessed for 7 days, this test quantified latency for mice to fall off an accelerating rotating rod. At 56 days old, mice were habituated for 3 days prior to testing. Training Day 1: Mice were placed on the stationary rod for 30 s. If mice fell, they were returned to the stationary rod until they could stay for 30 s. After 30 s, the rod rotated at a constant speed (5 rpm) so mice could learn to walk on the rotating rod. This trial was repeated until mice could walk for 15 s at 5 rpm without falling. Training Day 2 and 3: Rod rotation accelerated 1 rpm every 5 s until mice fell off. Following a fall, mice were returned to their home cage for 10 min to rest. The trial was then repeated a second time. Trials: Mice were tested at 1, 3, 5, and 7 days post-training, and consisted of 3 trials where mice were placed on the rod at a speed of 10 rpm accelerated 1 rpm every 5 s. The trial was repeated once if the animal fell within 5 s. Trials 1 and 2 were completed back-to-back to prevent associations between falling and returning to home cage. Animals were given a 10 min rest between trials 2 and 3. Mice were all placed facing forward at the beginning of the test and were not adjusted to face forward if they turned to face the wrong direction. Latency to fall was averaged for the best two trials per day to generate a time score for each mouse. Individual mouse scores were averaged in each group for each day, which precludes a RMANOVA analysis.

### Open field

This environment assessed locomotor behavior and anxiety, and habituated animals to the arena for NOR (see below). At 66 days old, mice were placed in the center of the arena (45 cm x 45 cm x 30 cm) and allowed 5 min to explore. Behavioral outcome measures included the total distance travelled (cm) and amount of time spent in the arena center (not thigmotaxic exploratory behavior).

### Novel object recognition (NOR)

NOR takes advantage of rodent’s natural curiosity for novelty in their environment [11, 41]. At 66 days old, mice continued in the Open Field arena for 60 min to complete habituation to the test arena. At 67 days old, two identical objects were placed in the arena, and mice were allowed to investigate for 5 min. The objects used for NOR were identical colored toys (Fisher-Price Little People, Mattel Inc, El Segundo, CA) placed in opposite corners in the arena with space behind the object for the mouse to observe it from all sides. The novel object was always a different color toy but with similar dimensions as the familiar object. Mice were returned to their home cage for a 4 h inter-trial interval before being returned to the arena, where one of the two original objects was replaced with a novel object. A discrimination ratio was calculated by dividing the amount of time mice explored the novel object by the total time spent exploring both objects, wherein 50% is considered chance.

### Elevated plus maze

The apparatus is constructed of four white acrylic platforms 5.1 cm wide x 30.5 cm long elevated 108.5 cm above the ground forming a cross in the center at 90 degree angles. Opposite one another, two platforms were enclosed with 15.2 cm high black opaque acrylic walls to decrease light exposure and increase seclusion, while the other two platforms had no walls. At 74 days old, mice were placed at the center cross and left to freely explore maze arms for 5 min before being returned to their home cage. The number of arm entries and time spent in open arms of the maze was recorded with Ethovision software.

### Y-Maze

This test assessed spatial navigation and measured memory of maze arms previously visited, as mice predominantly explore arms they have not recently visited. The apparatus is a white acrylonitrile butadiene styrene (ABS) plastic Y shaped maze with three 38.1 cm long and 7.6 cm wide arms with ABS plastic walls 12.7 cm high. The angle between each arm is 120°. At 82 days old, mice were placed in the center and allowed to explore each arm during a single 5 min trial. Scoring included sequential entry of all arms before entering another arm and total number of arm entries.

### Video recordings and aggressive behavior scoring

A CCTV mini surveillance camera (Vansxe, Cctv mini Sony Effio 2.8–12 mm varifocal lens box camera) was positioned at cage rear to continuously record behavior onto a surveillance DVR (Q-See - QC958-2 Digital Video Recorder in H.264 Format − 2 TB HDD). Recordings (B/W, 1920 × 1080/60i quality) were made in 7 h sessions based upon previous study [35] and began with 1 h remaining in the light cycle and continued 6 h into the dark cycle. Our previous study showed mice were inactive during the light cycle (except when personnel were present) and became active and overtly aggressive in the hour leading up to dark onset. Aggression increased when the dark cycle began (red light) and decreased over the next 6 h [35]. We chose to record behavior in the absence of interruptions from personnel or confounds from behavior testing.

Recordings occurred on days 133, 137, 151, 158, and 179, in conjunction with cage change schedules. Day 133 was between cage changes and served as baseline for aggressive behavior scoring. Day 137 provided data at cage change. Day 151 was a cage change that included the introduction or removal of the cage-divider, where the divider was removed from divided cages and a new divider was added to standard cages. Day 158 was between cage changes at 7 days following the introduction or removal of the cage-divider. Day 179 was between cage changes at 28 days following cage-divider introduction or removal. Experimental timeline is presented graphically (Fig. 1).

Two independent, blinded observers tallied each aggressive behavior in each cage (total 420 h). Observers were trained for two weeks prior to video assessment until inter-rater assessments were within 98% accuracy, measured using Cohen’s Kappa Statistic wherein the agreement rating was equivalent to near perfect agreement.

Aggressive behavior included: posturing (characterized by a tail rattle or thump, pouncing, sparring, mounting, barking, and chasing); scuffle/fight (two or more mice engaged in a scuffle (< 1 s) or an outright fight (≥ 1 s)); and unprovoked biting (one animal biting another without prior posturing or fighting). The number of aggressive events were summed and collated within each cage, averaged, and analyzed. We did not assess any other activity that occurred in the cage and did not utilize an activity budget.

### Weight and bite wounds

Animal weights can be used to assess animal health and well-being [23, 25, 26, 28]. Body weights were taken weekly for the first 10 weeks following weaning, and again during recording of home cage behavior (days 133–179). On each day of video recording, animals were inspected visually by animal care staff for bite wounds, 9 h prior to recording home cage behavior. The bite wound count per animal in each cage was recorded.

### Statistical analysis

A sample size estimation with an effect size of 0.86 was estimated based on preliminary studies and previous data for C57BL/6J mice for open field testing. To detect differences between groups, an estimated sample of 16 animals per group was necessary for behavior and a sample size of 5 cages (with 5 mice per cage) was estimated to detect differences in aggressive events and achieve β > 0.85 at α = 0.05. Therefore, 5 cages of 5 mice each (25 mice total per group, per condition) were used in this study.

Analysis of behavioral performance weight, aggressive events, and bite wounds were analyzed with SPSS (International Business Machines, Chicago, IL, v26). Statistical test outcomes are included in the results and table. For all data, the assumption that data were normally distributed was confirmed to ensure the validity of the statistical approaches used. Appropriate corrections were applied to any violations of homogeneity or sphericity. For instance, Maulchy’s sphericity test assesses violations of equal variances between all possible pairs of within subjects conditions [42]. If a violation occurred, an epsilon score was provided to measure the departure from sphericity and was corrected as follows: the Greenhouse-Geisser correction was used when ε was < 0.75 and the Huynh-Feldt correction when > 0.75.

Behavioral data were analyzed using an ANOVA with cage ID as a nested variable within the housing condition. A randomized block design ANOVA was used to analyze the rotarod data.

Individual mouse body weights were converted to z-scores to normalize the data and then subjected to a RMANOVA.

Number of aggressive events are shown as mean ± SEM. Number of aggressive events for each cage and total bite wounds per cage were analyzed between caging conditions using a RMANOVA followed by a Bonferroni post hoc analysis, with significance ascribed when *p* < 0.05, unless otherwise indicated.

## Results

### Behavioral testing

Standard neurological behavior tasks are well documented in C57BL/6J mice. As presented systematically here in the results, the series of motor, cognition, and anxiety tests conducted during the first 90 days showed that the expression of anxiety was statistically lower in mice reared in divided caging. None of the other behavioral tests showed significant differences between caging conditions. Behavioral test analyses included 25 mice from each condition for a total of 50 mice.

For motor performance on the rotarod, there was a significant main effect of caging condition, as mice in the divided cage condition had a 4.05 s longer trial on average across all training days compared to mice in the standard cage condition across all days (F(1,240) = 4.17, *p* < 0.05), and an expected main effect of training day as all mice performed better as training progressed (F(4,240) = 4.64, *p* < 0.05). However, there was no interaction of day by cage condition (F(4,240) = 0.67, *p* = 0.61; Table 1).

**Table 1:** Behavioral performance results for mice reared in partially divided compared to standard cages

		Divided	Standard	Test Statistics
Rotarod^†^	Duration All Days (s)	64.27 ± 3.59	60.22 ± 2.97	F_(1,240)_ = 4.17, p < 0.05^*^
	Time Effect (Δs)	10.24 ± 0.68	11.28 ± 0.98	F_(1,240)_ = 4.64, p < 0.05^*^
Open Field	Distance Traveled (cm)	1979.50 ± 155.39	2169.57 ± 120.89	F_(1,40)_ = 0.35, p = 0.57
	Time in Center (s)	12.01 ± 1.89	14.99 ± 1.93	F_(1,40)_ = 0.74, p = 0.41
Novel Object	Discrimination Ratio	0.49 ± 0.03	0.56 ± 0.03	F_(1,39)_ = 4.28, p = 0.07
Y-Maze	% Alternation	23.76 ± 1.74	24.32 ± 1.26	F_(1,40)_ = 0.04, p = 0.85
	Arm Entries	12.08 ± 0.93	13.00 ± 0.76	F_(1,40)_ = 0.33, p = 0.58
Elevated Plus Maze	Open Arm Entries	20.13 ± 2.52	13.04 ± 1.84	F_(1,39)_ = 2.74, p = 0.14
	Time in Open Arms (s)	33.98 ± 4.37	20.14 ± 3.22	F_(1,39)_ = 5.87, p < 0.05^†^

Behavioral data are shown as mean ± SEM. ^*^, indicates statistical difference between divided and standard caging at p < 0.05. ^†^, seconds

Open field revealed no significant differences in measurements collected. Analysis for distance traveled violated the assumption of homogeneity and was therefore corrected. There were no significant differences for distance traveled (cm) (F(1,40) = 0.35, *p* = 0.57), time in the center (F(1,40) = 0.74, *p* = 0.41), or percent of time exhibiting thigmotaxic behavior (F(1,40) = 1.44, *p* = 0.27; Table 1) [43]. 

Habituation to the testing arena for one hour served to reduce stress and promote evaluation of cognitive performance [43]. Performance in novel object recognition (discrimination ratio) was equivalent in mice from different caging conditions (F(1,39 = 4.28, *p* = 0.07), as mice spent similar time exploring the novel object (Table 1).

Performance in the Y-maze revealed no significant differences in spontaneous alternations (F(1,40) = 0.04, *p* = 0.85) or arm entries (F(1,40) = 0.33, *p* = 0.58) between caging conditions (Table 1).

**Fig. 3:**
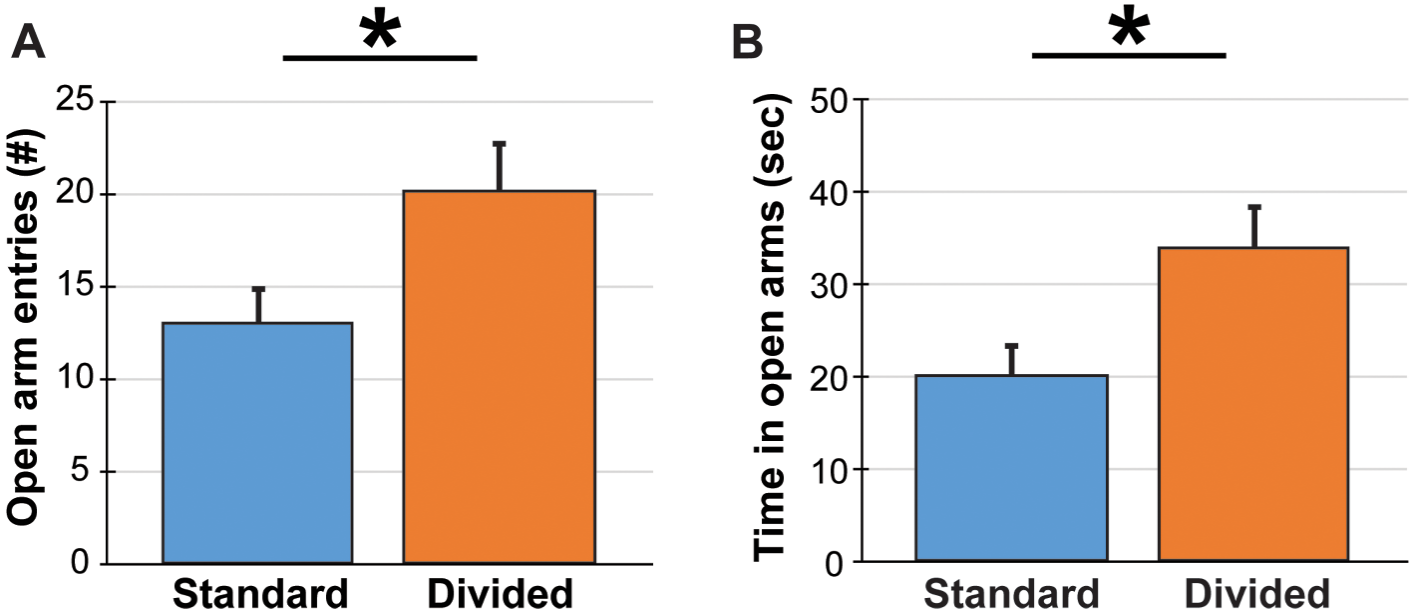
Elevated-plus maze measured anxiety of mice. Mice reared in divided caging had significant differences in the number of open arm entries (**A**) and time in the open arms of the arena (**B**) compared to standard caging controls. This outcome indicates more exploratory behavior and less anxiety. Data are graphed as mean ± SEM.

Anxiety-like behavior in the elevated plus maze revealed significant differences between caging conditions. There was no difference between mice from divided cages versus the standard cages for open arm entries (F(1,39) = 2.74, *p* = 0.14; Fig. 3A). However, mice in divided cages spent more time in the open arms (F(1,39) = 5.87, *p* = 0.04; Fig. 3B), indicating less anxiety and more exploratory behavior. One animal was excluded for jumping off the apparatus.

### Body weight

**Fig. 4:**
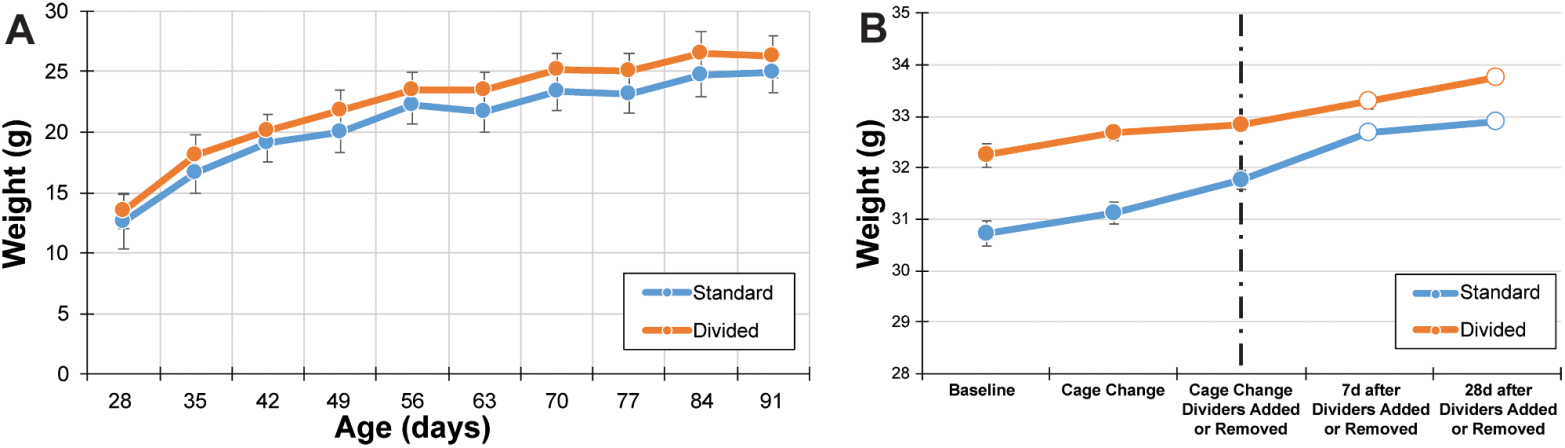
Comparison of mouse body weight between standard and divided caging, beginning at 28 days old (**A**). Over 10 weeks, body weight increases are consistent with upper limit thresholds for this strain as made available by The Jackson Laboratory. By repeated measure ANOVA, body weight has a main effect of time and condition (see results). (**B**) Body weight was lower for mice housed in standard caging until the addition of a cage divider. Open dots indicate body weights following the introduction or removal of a cage divider. Dashed line indicates the cage change time point when the cage divider was introduced or removed from standard or divided caging, respectively

Mice reared in divided caging weighed more than mice reared in standard caging over time. Mauchly’s test indicated that the assumption of sphericity had been violated (*χ*^2^(44) = 912.74, *p* = 0.00), therefore degrees of freedom were corrected using the Greenhouse-Geisser estimates of sphericity (*ε* = 0.130). The RMANOVA revealed a significant main effect over time (F(1.17, 56.26) = 76.69, *p* < 0.05), a main for caging condition (F(1, 48) = 6.32, *p* < 0.05), as well as an interaction between time and caging condition (F(1.17, 56.26) = 6.14, *p* < 0.05; Fig. 4A).

Individual weights were recorded while conducting the aggression analysis, which began at day 133, and ended on day 179 (Fig. 4B). Mauchly’s test indicated that the assumption of sphericity had been violated (χ^2^(9) = 106.36, *p* = 0.00), therefore degrees of freedom were corrected using the Greenhouse-Geisser estimates of sphericity (ε = 0.44). The RMANOVA revealed a within subjects main effect of time (F(1.76, 84.56) = 57.12, *p* < 0.05) and an interaction between time and caging condition (F(1.76, 84.56) = 3.34, *p* < 0.05), but not a main effect for caging condition (F(1, 48) = 1.18, *p* = 0.19).

### Aggressive events

**Fig. 5:**
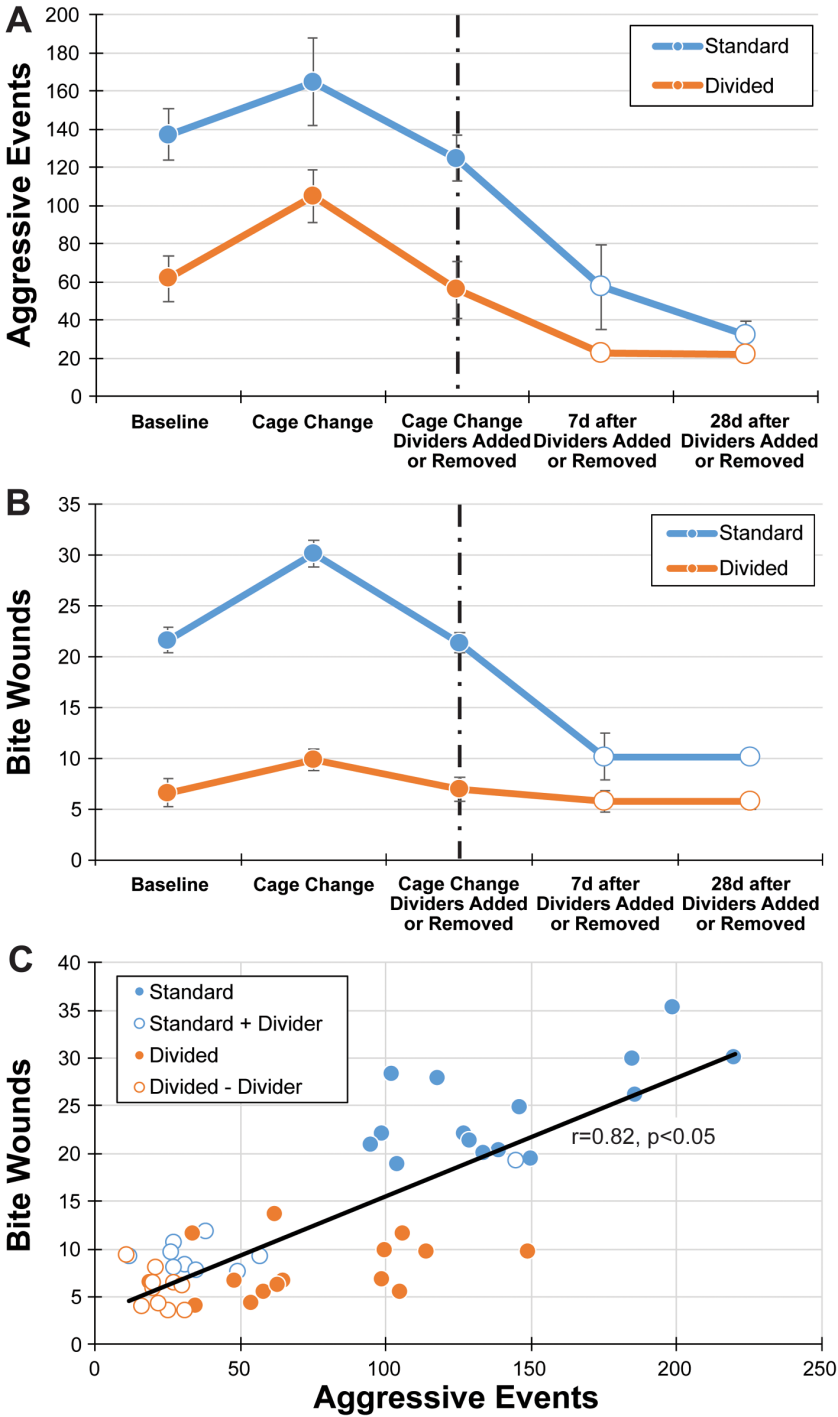
Divided caging had significantly fewer aggressive behavior events than standard caging (**A**), and significantly fewer bite wounds than standard caging (**B**). Open dots indicate bite wounds following the introduction or removal of a cage divider. Dashed line indicates the cage change time point when the cage divider was introduced or removed from standard or divided caging, respectively. (**C**) The correlation between the number of aggressive events and bite wounds in a cage was positive and significant. Individual cage conditions (standard: blue; divided: orange) and time points (housing: filled circle; after addition (+) or removal (-) of a cage divider: open circle) are uniquely identified

Mice reared in divided caging had fewer total aggressive events than mice in standard caging over time. As expected, a planned cage change increased aggressive events compared to a baseline measurement recorded at the midpoint between cage changes (Fig. 5A). The observed aggressive events primarily included posturing and scuffling, with few unprovoked biting events. Introduction of a cage divider to standard caging and removal of the divider from divided caging evaluated the stability of the cage hierarchy and an intervention to reduce existing aggression, respectively. Divided caging maintained low levels of aggressive events in the absence of a divider. Standard caging benefitted from the addition of a divider, as indicated by reduced numbers of aggressive events. Statistical analysis indicated a violation of sphericity (χ²(14) = 44.970, *p* < 0.05), therefore degrees of freedom were corrected using Greenhouse-Geisser estimates of sphericity (ε = 0.37). The RMANOVA revealed a main effect across five observation points (F(1.86, 14.89) = 16.90, *p* < 0.05) and a main effect of caging condition (F(1, 8) = 31.23, *p* < 0.05), but no significant interaction between observations and caging condition (F(1.86, 14.89) = 1.756, *p* = 0.20).

### Bite wounds

Individual mice reared in divided caging had a negligible number of bite wounds at each observation point. The summed total number of bite wounds per standard cage was 4-fold higher than divided cages (Fig. 5B). With a cage change, the number of bite wounds increased further in the standard cages than the divided cages. Removal of the cage divider maintained a low total number of bite wounds. Standard caging benefitted from the addition of a divider, as indicated by reduced total number of bite wounds. Statistical analysis indicated a violation of sphericity (χ²(9) = 30.51, *p* < 0.05), therefore degrees of freedom were corrected using Greenhouse-Geisser estimates of sphericity (ε = 0.38). The RMANOVA was performed on the total number of bite wounds per cage each observation point and revealed a main effect of observation points (F(1.54, 12.29) = 89.06, *p* < 0.05) and a main effect of caging condition (F(1, 8) = 274.03, *p* < 0.05) as well as a significant interaction between observations and caging condition (F(1.54, 12.29) = 28.89, *p* < 0.05).

Total bite wounds correlated with the total number of aggressive events. A Pearson’s correlation between aggressive events (video recording) and bite wounds (visual inspection) showed a significant positive correlation (*r* = 0.82, *p* < 0.05; Fig. 5C), indicating that standard welfare checks for bite wounds can be applied to evaluate prior aggression and the efficacy of an intervention to control aggression.

## Discussion

Cage dividers provide a complex housing condition for laboratory mice that may better resemble their natural habitat [12, 25, 37]. We investigated the impact of partial cage dividers on behavioral performance, weight, aggressive behavior, and biting. Weaning non-sibling mice into divided or standard caging did not adversely affect performance in standard neurological tasks. Further, divided caging had long-term effects on anxiety behavior and weight, in addition to significantly attenuating aggressive behavior in group-housed C57BL/6J male mice. The late addition of partial cage dividers to standard caging reduced aggression between mice. These results indicate that animal welfare and research may be improved with the use of partial cage dividers.

We report mouse standard behavioral tests, body weight data, and home-cage aggression over 180 days under experimental conditions of different home cage conditions. These data represent evaluations during the standard growth of the mice, and then young adult through adult lifespan. Mice reared in divided cages weighed more than those reared in standard cages, which is consistent with reports of complex cage design studies [7, 12, 25, 31, 35] and indicative of healthier animals [9]. For future analysis, body weight variability within a cage and between caging conditions may estimate the breadth of dominance hierarchies and individual access to food/water. Animal appearance remained unremarkable for the duration of the study and, aside from bite wounds that did not require veterinary intervention, no other adverse events or veterinary concerns were reported.

Performance on standard behavior tasks indicated that motor and cognitive performance met normal expectations for both caging conditions. The motor performance on the rotarod also showed adaptation to training. A significant decrease in anxiety at 70 days old was associated with more time in the open arms of the elevated plus maze by mice reared in partially divided caging. Thus, housing mice in partially divided caging will not significantly divert locomotor or cognitive behavioral performance and will reduce anxiety in mice. Since mouse behavior covaries with stress, mice housed with partial cage dividers may habituate faster to animal handling and behavioral arenas [43]. Beyond behavior, modified housing conditions could reduce variability in physiological outcomes of inflammation and stress-related hormones [44]. Future investigations can determine the effects of divided caging on physiological outcomes.

As expected, non-sibling, group housed, male mice were aggressive towards one another. Aggressive behaviors within a cage were quantified at 133 days old as video recorded aggressive encounters and the number of bite wounds per cage. At this point, mice in standard caging showed 2-fold increases in aggression rates and 4-fold increases in bite wounds compared to mice in divided caging. These observations are interpreted to represent the established dominance hierarchy within a cage, which was established early and maintained; aggressive behavior was not specifically recorded prior to 133 days old. The addition of cage dividers to long-established cage conditions decreases aggressive events and bite wounds, while removing dividers from long-established cages did not increase either aggressive events or bite wounds. Cage dividers help to establish a functional hierarchy in laboratory mouse cages, either at weaning or at later stages. Further, bite wound counts directly correlated with laborious video-observed aggressive event data, suggesting that bite wound counts can serve as a rapid surrogate measure for aggressive events. Relying on bite wounds may be preferable for repeated, routine evaluation, whereas video, dermatological analysis, and post-mortem skin histology would be reserved to test specific hypotheses.

Here we extend the investigation into the impact of partial cage dividers on neurobehavioral performance and aggressive events. The partial cage division is accomplished by a corrugated plastic insert that divides half of the cage in thirds. The plastic insert was designed as part of the caging condition, similar to the shoebox and feed hopper; as such the divider was neither cleaned nor replaced. Cage dividers of other material may require sterilization, replacement, or other routine upkeep. Further, the partial cage dividers are distinct from grid cage dividers that bisect a cage for pair housing. Grid dividers prevent physical contact between mice, while allowing sight and smell [45–47]. Pair housed mice require separate food, water, and nesting sources, unlike the shared resources in a partially divided cage [45–47]. Under pair-housed, but separate, conditions, mice show combinations of impaired nest building, lower body weight, increased heart rate, and elevated stress responses [45–47]. The interpretation remains that a grid divider prevents the expression of submissive behavior (e.g., break eye contact), and yet the individual burrow width in the partially divided cage was not observed as an ambush point to express dominance.

Weaning animals into divided caging added complexity [7, 12, 25, 26] and routes of retreat [12, 25], thereby enabling the expression of proper social cues [3, 6, 7, 22, 23, 31, 48]. To this end, partial cage dividers helped to establish a functional cage hierarchy as expressed by reductions in aggressive events, bite wounds, and anxiety [4, 5, 17, 19, 20, 48]. Long-term housing in divided caging established a cage hierarchy that was maintained once the divider was removed, as evidenced by an absence of increased aggressive behavior. Age could have played a role in decreased aggression, but was not observed in the standard caging group. Also, weight change and atypical behavior can result from continued stressors of a dysfunctional hierarchy, fighting, or isolation, which would negatively impact research reproducibility and scientific rigor [33, 38].

Reductions in aggression as a result of aging was not explored, which would require continuous housing under the same conditions. Here we report results for wildtype mice weaned into the divided cages, where the impact of cage dividers on breeding colonies and trans-generational use remains to be determined. Adding physiological measures, such as fecal microbiome and serum corticosterone, may expand understanding of cage conditions on overall animal welfare.

While aggression and social hierarchy continue to be studied [20] with focus on neuronal organization and circuit components of behavior, the potential of partial cage dividers to reduce aggression and improve overall animal welfare demands continued evaluation. Results are indicated for C57BL/6J mice and may not translate to all strains. Additionally, there are multiple research conditions (breeding, generational breeding, neurodegenerative or injury models, etc.) that may also benefit from divided cages. Partial cage dividers provide one possible solution to prevent or mitigate aggression observed in group-housed male mice. Opportunities for laboratory mice to express natural behaviors can minimize abnormal behavior and thereby enhance scientific rigor. Given the potential welfare benefits, partial cage dividers could be considered a viable option for behavioral management programs.

The partial cage dividers used in this study were hand-crafted specifically for use in the Innovive™ mouse caging system. The concept of partial cage division – several burrows and a common area – can be adapted to any caging system. The critical design features include floor to cage top vertical walls held in place by the lid that create the burrows and common area. The choice of construction materials would depend on standards of practice regarding sterilization, cleaning, and durability. One primary consideration is the effort necessary to produce the anticipated number of dividers for a specific study, rack, or facility.

## Data Availability

Data are available upon reasonable request to the corresponding author.
